# Awareness, adoption, and application of the Association of College & Research Libraries (ACRL) Framework for Information Literacy in health sciences libraries^*^

**DOI:** 10.5195/jmla.2017.131

**Published:** 2017-10-01

**Authors:** Stephanie J. Schulte, Maureen Knapp

## Abstract

**Objective:**

In early 2016, the Association of College & Research Libraries (ACRL) officially adopted a conceptual Framework for Information Literacy (Framework) that was a significant shift away from the previous standards-based approach. This study sought to determine (1) if health sciences librarians are aware of the recent Framework for Information Literacy; (2) if they have used the Framework to change their instruction or communication with faculty, and if so, what changes have taken place; and (3) if certain librarian characteristics are associated with the likelihood of adopting the Framework.

**Methods:**

This study utilized a descriptive electronic survey.

**Results:**

Half of all respondents were aware of and were using or had plans to use the Framework. Academic health sciences librarians and general academic librarians were more likely than hospital librarians to be aware of the Framework. Those using the Framework were mostly revising and creating content, revising their teaching approach, and learning more about the Framework. Framework users commented that it was influencing how they thought about and discussed information literacy with faculty and students. Most hospital librarians and half the academic health sciences librarians were not using and had no plans to use the Framework. Librarians with more than twenty years of experience were less likely to be aware of the Framework and more likely to have no plans to use it. Common reasons for not using the Framework were lack of awareness of a new version and lack of involvement in formal instruction.

**Conclusion:**

The results suggest that there is room to improve awareness and application of the Framework among health sciences librarians.

## INTRODUCTION

The Association of College & Research Libraries (ACRL) Framework for Information Literacy (hereafter referred to as the “Framework”) [1] was officially adopted by the ACRL board in January 2016 after a two-year process of drafting and incorporating extensive feedback from library professionals. Given significant changes in the information landscape since the adoption of the 2000 ACRL Information Literacy Competency Standards [2] (hereafter referred to as the “Standards”), the Framework grew out of a need to bring the existing Standards into alignment with modern information creation and use. The process of adopting the Framework and rescinding the Standards has been controversial. While proponents praise the Framework for its flexibility [3], others have criticized it as elitist [4]. Regardless, the changes to information literacy (IL) standards have brought about renewed energy and a new level of discussion surrounding instruction among academic librarians.

What exactly are the changes to IL standards? The main difference is a shift from observable behavioral standards to a more philosophical theory of threshold concepts. While the Standards outlined specific task-based learning objectives (e.g., “accesses needed information effectively,” “evaluates information and its sources critically”) [2], the Framework introduces “a cluster of interconnected core concepts,” (e.g., “Authority Is Constructed and Contextual,” “Scholarship as Conversation”) [1]. Threshold concepts “are those ideas in any discipline that are passageways or portals to enlarged understanding or ways of thinking and practicing within that discipline” [1]. The assessment of information-literate behaviors has also shifted. Whereas the Standards offered a list of twenty-two measurable performance indicators for IL, the Framework shies away from any “prescriptive enumeration of skills” [1] and encourages librarians to develop their own methods of assessment based on their needs.

While discussions of ACRL IL standards are relevant to general academic librarians, health sciences librarians often work outside ACRL directives because they are obligated to follow discipline-specific accreditation standards and learning objectives. Many health sciences librarians teach within the evidence-based practice (EBP) paradigm rather than strictly teach IL. Several authors have compared EBP to IL [5–7] and have described integrating IL into health sciences instruction [8–12]. However, the authors speculate that librarian-instructors in the health sciences do not regularly consult ACRL resources to inform their teaching practices.

Because of the emphasis on discipline-specific learning standards as well as the newness of the Framework, little is known about its application by health sciences librarians. Knapp and Brower wrote about how the Framework might affect instruction by health sciences librarians, speculating that the Framework’s focus on knowledge-based search and discovery concepts (rather than discrete skills outlined in the Standards) addresses the information needs of upper-level researchers, while its conceptual flexibility allows health sciences librarians to address learning at all levels of scholarly development [13]. While plenty of published literature describes the use of either IL concepts or the congruent EBP paradigm to inform teaching strategies, to date, only one case study incorporates threshold concepts and discusses the Framework in relation to instruction by health sciences librarians. Alpi and Hoggan examined threshold concepts and described a “typology of transformational learning outcomes” in a veterinary medicine summer research program [14]. They developed threshold concepts by drawing from IL, health professions, and veterinary research literature and described alignment of the threshold concepts with content in the research program.

The purpose of this descriptive study was to assess awareness of the Framework among health sciences librarians and to determine the level of adoption and application of the Framework in instruction by health sciences librarians. The timing of the study represented awareness, adoption, and application of the Framework at an early point in its existence, approximately one year after its initial filing with ACRL and just prior to its official adoption and ACRL’s subsequent rescinding of the previous Standards. Specifically, the study aimed to determine (1) whether health sciences librarians were aware of the new Framework; (2) whether they had used the Framework to change their instruction or communication with faculty and, if so, what changes had taken place; and (3) whether certain librarian characteristics were associated with the likelihood of adopting the Framework.

## METHODS

This study was declared exempt from review by both authors’ university institutional review boards. We distributed a survey electronically via the Qualtrics platform. The target sample was health sciences librarians in general academic, academic medical center or medical school, and hospital library settings. We used health sciences library email lists to recruit participants, including MEDLIB-L, regional lists of the National Network of Libraries of Medicine, and Medical Library Association (MLA) chapters and sections. Because of significant overlap of email subscribers, the final size of the targeted sample was unknown.

The survey (supplemental appendix) consisted of sixteen multiple choice questions, with options to add comments. One question was a free essay response. Skip logic was enabled to reveal more questions depending on a respondent’s answers to certain questions. The survey remained open for thirty days during January and February 2016 with a reminder email sent approximately two weeks after the initial recruitment email. Data were analyzed using descriptive statistics as well as cross-tabulations and chi-square tests of pairs of questions.

Chi-square analyses and Fisher’s exact tests (FET) (for distributions with expected counts less than 5, using Freeman-Halton extension, if needed) using a 95% confidence level were conducted to determine the statistical significance of relationships between awareness and use of the older Standards or the new Framework. For the purposes of analyses, the categories of “no” and “not sure” were combined for any question with this option, as were the categories of “yes” and “no, but I plan to use it soon” for the use of the Framework question only.

For this analysis, we collapsed years of experience into increments of 10 years (0–10 years, 11–20 years, greater than 20 years). Missing data were not included in statistical analyses. Since the vast majority of respondents were members of a professional organization and identified instruction as a job role (97.5% and 90.0%, respectively), chi-square analyses were not conducted to examine whether membership in a professional society or instruction as a work responsibility were related to awareness and use of the Standards or Framework.

## RESULTS

Table 1 provides details about participant characteristics. Out of 146 respondents, 130 answered at least some of the questions, and 120 respondents completed the survey. Three out of 120 chose to skip some questions in the middle of the survey. Respondents were geographically diverse and represented a spectrum of newer librarians (22% with less than 5 years of experience) to very experienced librarians (34% with more than 20 years of experience). The majority of respondents (62%) were from academic health sciences libraries. Hospital and general academic libraries were also represented (24% and 14%, respectively). Nearly all (97%) respondents reported being members of professional organizations, with 84% noting membership in MLA, 77% noting membership in their regional MLA chapter, and 53% noting membership in a state or regional library association. About a quarter of respondents were members of the American Library Association (ALA, 28%) or ACRL (24%).

**Table 1:** Participant demographics^*^

**Demographics**	**n**
Years worked in libraries	119
<5 years	26
6–10 years	22
11–15 years	13
16–20 years	17
>20 years	41
Type of library	119
Academic health sciences	74
Hospital	28
General academic	12
Other	5
Membership in professional organizations	
Yes	116
Medical Library Association (MLA)	98
American Library Association (ALA)	33^†^
Association of College and Research Libraries (ACRL)	28
Regional chapter of MLA	89
State or regional library association	62
Special Libraries Association	11
Association for Information Science and Technology	3
No membership	3
Provision of instruction	
Provides instruction	109
One-on-one consultations	103
Course-integrated (e.g., one-shot or multiple sessions, online or face-to-face in another faculty member’s course)	83
Standalone workshops or continuing education	71
Credit-bearing courses (face-to-face or online)	19
Does not provide instruction	12
Time dedicated to planning/teaching in position	107
<25%	67
26%–50%	28
51%–75%	11
>75%	1
Membership in curriculum committee outside of library	
Yes	30
No	90

* Total values vary due to missing data.

† Five participants noted membership in ACRL but not ALA; thus, the total number of ALA members should likely be 38 given that ALA membership is required to join ACRL.

The vast majority of respondents (92%) provided instruction to students, faculty, or staff at their institution. The most common form of instruction was one-on-one consultation (94%), followed by course-integrated sessions provided in person or online (76%). One quarter of respondents reported serving on a curriculum committee external to the library.

Out of 130 participants who responded to questions about familiarity with the older Standards and the new Framework, 71% were familiar with the older Standards, but only 40% of these respondents had actually used the Standards for library instruction. A lower percentage (52%) were familiar with the new Framework. Of 128 respondents, only 14 (11%) indicated they were using the Framework in education or instruction efforts, while 45 (35%) were not using it but had plans to use it soon, and 69 (54%) were not using it and had no plans to use it. None of the 14 participants using the Framework were hospital librarians. The most common reasons for not planning to use the Framework were irrelevance to audience or typical instructional settings and unfamiliarity with the Framework.

Comments about unfamiliarity with the Framework were apparent across all types of librarians who participated. One academic health sciences librarian stated, “Wasn’t aware of it until now, and I can’t plan on using it until I look at it and decide it makes sense for my classes,” which was fairly representative of those who were unaware of the Framework. A hospital librarian similarly commented, “Just became aware of it. Have to learn what it is all about before I can use it.” Hospital librarians commented more frequently about irrelevance, citing, “My library instruction is generally one on one and more informal,” as well as, “I don’t teach classes.”

We also asked for qualitative comments about how the Framework was changing instruction or communication practices. Several respondents mentioned they were still in an investigative phase and figuring out how to use the Framework. Others commented that the Framework could potentially provide a broader lens for discussing IL and an avenue for more participatory instruction. One participant expressed the effect of the frame “authority is constructed and contextual” on their approach to discussing primary source selection, while another mentioned the “scholarship is a conversation” frame as a practice-changing phenomenon. Another participant said that they would continue to use the nursing-specific ACRL standards instead of the Framework.

Other criticisms included that the Framework was hard to understand and apply and that it used too much educational jargon. One comment hinted that some health sciences librarians felt that the Framework was not applicable unless they were specifically teaching IL: “Because I am not teaching traditional IL classes I don’t have a need to change anything about the way I am teaching or the assignments I am giving out.”

Table 2 details awareness and use of both the older Standards and the new Framework by type of library, years of experience, and average time spent teaching. Academic health sciences and general academic librarians were more likely than hospital librarians to be aware of both the Standards (*p*<0.05; FET) and the Framework (*p*<0.05; FET) and were more likely to have used or have plans to use the Standards (*p*<0.05; FET) and Framework (*p*<0.05; FET). In fact, the type of library where librarians were working was the only significant factor related to use of the Framework.

**Table 2:** Relationship between awareness and use of the ACRL Standards and Framework to type of library, years of experience, and time dedicated to teaching

	Awareness of Standards^*^				Use of Standards†				Awareness of Framework‡				Use of Framework§					
			
	Yes		No/not sure		Yes		No/not sure		Yes		No/not sure		Yes		No, but plan to		No plans to use	
								
	%	(n)	%	(n)	%	(n)	%	(n)	%	(n)	%	(n)	%	(n)	%	(n)	%	(n)
Type of library																		
Academic health sciences	49.6%	(59)	12.6%	(15)	29.4%	(35)	32.8%	(39)	37.8%	(45)	24.4%	(29)	6.8%	(8)	22.2%	(26)	32.5%	(38)
General academic	9.2%	(11)	0.8%	(1)	6.7%	(8)	3.4%	(4)	9.2%	(11)	0.8%	(1)	4.3%	(5)	5.1%	(6)	0.8%	(1)
Hospital	9.2%	(11)	14.2%	(17)	2.5%	(3)	21%	(25)	4.2%	(5)	19.3%	(23)	—	(0)	6%	(7)	17.9%	(21)
Other	3.4%	(4)	0.8%	(1)	1.7%	(2)	2.5%	(3)	1.7%	(2)	2.5%	(3)	0.8%	(1)	1.7%	(2)	1.7%	(2)
Years of experience																		
0–10	32%	(38)	8.4%	(10)	16.8%	(20)	23.5%	(28)	26%	(31)	14.3%	(17)	6%	(7)	15.4%	(18)	18.8%	(22)
11–20	21%	(25)	4.2%	(5)	12.6%	(15)	12.6%	(15)	16%	(19)	9.2%	(11)	4.3%	(5)	9.4%	(11)	12%	(14)
>20	18.5%	(22)	16%	(19)	10.9%	(13)	23.5%	(28)	10.9%	(13)	23.5%	(28)	1.7%	(2)	10.3%	(12)	22.2%	(26)
Average time spent teaching																		
≤25%	40.2%	(43)	22.4%	(24)	15.9%	(17)	46.7%	(50)	28%	(30)	34.6%	(37)	5.7%	(6)	21.7%	(23)	35%	(37)
26%–50%	23.4%	(25)	2.8%	(3)	19.6%	(21)	6.5%	(7)	18.7%	(20)	7.5%	(8)	4.7%	(5)	10.4%	(11)	11.3%	(12)
51%–75%	9.3%	(10)	0.9%	(1)	7.5%	(8)	2.8%	(3)	6.5%	(7)	3.7%	(4)	2.8%	(3)	3.8%	(4)	3.8%	(4)
>75%	0.9%	(1)	—	(0)	0.8%	(1)	—	(0)	0.9%	(1)	—	(0)	—	(0)	0.9%	(1)	—	(0)

* *p*<0.05 for type of library, years of experience, and average time spent teaching.

† *p*<0.05 for type of library and average time spent teaching.

‡ *p*<0.05 for type of library, years of experience, and average time spent teaching.

§ *p*<0.05 for type of library.

Librarians with fewer years of experience were more likely to be aware of both the Standards (χ^2^=9.84, *df*=2, *p*<0.05) and Framework (χ^2^=11.33, *df*=2, *p*<0.05); however, there were no statistically significant differences in use of the Standards and Framework based upon years of experience. Librarians who spent more time teaching in their jobs were more likely to be aware of the Standards (χ^2^=8.67, *df*=2, *p*<0.05) and Framework (χ^2^=6.5, *df*=2; *p*<0.05) and were more likely to have used the Standards (χ^2^=25.04, *df*=2, *p*<0.05). Also, librarians who were members of curriculum committees outside of the library were more likely to use the Standards (χ^2^=9.07, *df*=1, *p*<0.05), although this did not hold true for use of the Framework.

## DISCUSSION

We examined health sciences librarian awareness and use of the ACRL Framework at an early stage of the Framework’s adoption. We found that those working in academic health sciences and general academic libraries were more aware of both and had used the older ACRL Standards and new Framework more than hospital librarians had, although more than half of all participants had not used either. Librarians with a decade or less of experience and those who spent more time planning and conducting instruction were also more aware of the Framework. Based on comments from hospital librarians, lack of usage might be due to not recognizing how the Standards or the Framework fit in their typical roles, especially if the bulk of their teaching is actually one-on-one instruction through reference and research consultations or clinical rounds situations. Academic health sciences librarians might also fail to see the relevance of the Framework to their typical teaching settings.

The generalizability of our results might be limited. Participants self-selected via recruitment through various health sciences librarian email lists. It was possible that those who completed the survey were more likely to complete it because they had an existing interest in IL, especially as recruitment emails explained that the purpose of the survey was to gauge awareness and adoption of the Framework. Because of this, it was possible the results underestimated the lack of awareness and use of the Standards and Framework.

Our results suggest that librarians of all types are still determining how to incorporate into their instruction the broader approach that the new Framework promotes. Some librarians feel the Framework is not relevant to their typical instructional settings or to the audiences to whom they typically provide instruction. One possible reason is that some librarians simply do not recognize the parallels between IL and EBP, even though as Adams points out, they share the abilities to find, evaluate, and use information efficiently for a specific purpose [5].

Another reason health sciences librarians may not appreciate the applicability of the Framework could include the constraints of typical health sciences librarian instructional efforts. In a recent systematic review of instructional methods that health sciences librarians used to teach EBP [15], nearly all of the included studies had clinician or researcher coauthors, suggesting librarians often work with teams of faculty to integrate EBP. The compromises required when working in a faculty team where curricula are already crowded may limit health sciences librarians’ abilities to significantly change their approaches.

Health sciences librarians can take several steps to develop their understanding of the Framework to benefit their instructional efforts. Most importantly, health sciences librarians should read the Framework, which could lead to the recognition that at least three of the frames closely relate to EBP: authority as constructed and contextual, research as inquiry, and searching as exploration. The knowledge practices and dispositions that are included with each frame provide both practical learning outcomes that can assist with content development and behavioral characteristics of information literate individuals to fuel ideas for assessment. After carefully reading the Framework, health sciences librarians should take some of the suggestions provided in the Framework’s appendixes, including forming a group to discuss the concepts, reaching out to those served to have a discussion, piloting new approaches, and sharing successes and failures [1]. Additionally, ACRL has developed a publicly accessible sandbox in which librarians can find many examples of activities developed by other library professionals [16].

Another step is to seek out professional development on the topics of instruction and instructional design, especially those explicitly connected to the Framework. MLA offers an on-demand webinar series on instructional design, which, although not directly connected to the Framework, addresses many features of effective teaching that work hand-in-hand with the ideas of the Framework. Health sciences librarians can also look to institutional teaching and learning centers, library professional organizations, or regional National Network of Libraries of Medicine offices for workshops about instructional approaches or express interest in these types of workshops, if they are not yet available. Becoming more fluent in the language of instructional design can also serve to empower librarians to speak more authoritatively when designing instruction.

Grappling with the application of the Framework is quite possibly creating a state of liminality for many librarians, a condition that Meyer, Land, and Baillie describe as a “suspended state of partial understanding or ‘stuck place’” that is necessary for crossing a learning threshold but can also be simultaneously troubling [17]. Badke describes the stresses and strains shared by many and in response to critics of the Framework notes, “Maybe the reason why IL is so marginal in the academic world after all these years is that [it] has not been properly expressed as essential to the scholarly enterprise” [18]. He suggests—and we agree—that the Framework may be, at least in part, an antidote to this problem.

## SUPPLEMENTAL FILES

Appendix Survey
