# Supplementary material for: Awareness, adoption, and application of the Association of College & Research Libraries (ACRL) Framework for Information Literacy in health sciences libraries
Source: J Med Libr Assoc. 2017 Oct 1;105(4):347–54. doi: 10.5195/jmla.2017.131 (PMC5624424; doi:10.5195/jmla.2017.131)
Supplement: Appendix [file jmla-105-347-s001.pdf]

JMLA Journal of the Medical Library Association

SC
Supplemental content to
J Med Libr Assoc. Oct;105(4): dx.doi.org/10.5195/jmla.2017.131
www.jmla.mlanet.org
© Schulte; Knapp 2017

Awareness, adoption, and application of the Association of College and Research Libraries (ACRL) Framework for Information Literacy in health sciences libraries

Stephanie J. Schulte; Maureen Knapp, AHIP

APPENDIX

Survey

1. Are you familiar with the Association of College & Research Libraries' (ACRL) Information Literacy Competency Standards for Higher Education (Chicago, 2000)?
☐ Yes (1)
☐ No (2)
☐ Not sure (3)

2. Have you used the ACRL's Information Literacy Competency Standards for Higher Education for library instruction activities in the past?
☐ Yes (1)
☐ No (2)
☐ Not sure (3)

3. Are you aware of the recently released Framework for Information Literacy for Higher Education (Chicago, 2015)?
☐ Yes (1)
☐ No (2)
☐ Not sure (3)

4. Are you using the Framework for Information Literacy for Higher Education (2015) in any of your education or instruction efforts?
☐ Yes (1)
☐ No, I have no plans to use it at this time (2)
☐ No, but I plan to use it soon (3)
If No, I have no plans to use it at this time is selected, skip to question #7.

5. How are you using or how do you plan to use the Framework? Choose all that apply.
☐ Learning more about the Framework (1)
☐ Mapping the Framework to discipline-specific competencies (2)
☐ Creating or revising content/learning objectives for instruction sessions/library classes (3)
☐ Revising the teaching method/approach used in instruction sessions/library classes (4)
☐ Creating or revising assignments or activities for use in instruction sessions/library classes (5)
☐ Revising a discipline-specific curriculum to reflect changes (6)
☐ Notifying educational stakeholders at institution about changes (7)
☐ Other (please describe) (8) ____________________

6. Please describe how the Framework has or is changing your instruction and/or your communication with faculty, staff, and students.

____

JMLA Journal of the Medical Library Association

SC
Supplemental content to
J Med Libr Assoc. Oct;105(4): dx.doi.org/10.5195/jmla.2017.131
www.jmla.mlanet.org
© Schulte; Knapp 2017

7. Why don't you plan to use the Framework in your education or instruction efforts?
- ☐ Not relevant to my instruction efforts (1)
- ☐ Not relevant to my typical audience (2)
- ☐ Too difficult to understand (3)
- ☐ Prefer the approach and language of the Information Literacy Competency Standards for Higher Education (4)
- ☐ Other (please describe) (5) ________________________________

8. Do you personally provide instruction to students, faculty, or staff at your institution?
- ☐ Yes (1)
- ☐ No (2)

If no, skip to question # 11.

9. How do you provide instruction to students, faculty, or staff at your institution? Select all that apply.
- ☐ Course-integrated instruction, such as one-shot sessions, multiple sessions, or online videos or modules integrated into another faculty member's course (online or face-to-face) (1)
- ☐ Credit-bearing course(s) (online or face-to-face) (2)
- ☐ Stand-alone workshops, including continuing education sessions (online or face-to-face) (3)
- ☐ One-on-one at the reference desk or by individual consultation (4)
- ☐ Other (please describe) (5) ________________________________

10. On average, how much time do you spend in total planning instruction and actually teaching as part of your current position?
- ☐ Up to 25% (1)
- ☐ 26%–50% (2)
- ☐ 51%–75% (3)
- ☐ More than 75% (4)

11. Are you a member of a curriculum committee outside of the library at your institution?
- ☐ Yes (1)
- ☐ No (2)

12. What is your geographic region?
- ☐ Greater Midwest region (Illinois, Indiana, Iowa, Kentucky, Michigan, Minnesota, North Dakota, Ohio, South Dakota, Wisconsin) (1)
- ☐ MidContinental region (Colorado, Kansas, Missouri, Nebraska, Utah, Wyoming) (2)
- ☐ Middle Atlantic region (Delaware, New Jersey, New York, Pennsylvania) (3)
- ☐ New England region (Connecticut, Maine, Massachusetts, New Hampshire, Rhode Island, Vermont) (4)
- ☐ Pacific Northwest region (Alaska, Idaho, Montana, Oregon, Washington) (5)
- ☐ Pacific Southwest region (Arizona, California, Hawaii, Nevada, US territories in the Pacific) (6)
- ☐ South Central region (Arkansas, Louisiana, New Mexico, Oklahoma, Texas) (7)
- ☐ Southeastern Atlantic region (Alabama, District of Columbia, Florida, Georgia, Maryland, Mississippi, North Carolina, Puerto Rico, South Carolina, Tennessee, US Virgin Islands, Virginia, West Virginia) (8)
- ☐ Canada (9)
- ☐ Other (please describe) (10) ________________________________

JMLA Journal of the Medical Library Association

SC

Supplemental content to
J Med Libr Assoc. Oct;105(4): dx.doi.org/10.5195/jmla.2017.131
www.jmla.mlanet.org
© Schulte; Knapp 2017

13. How many years have you worked in libraries?
- ☐ 0–5 years (1)
- ☐ 6–10 years (2)
- ☐ 11–15 years (3)
- ☐ 16-20 years (4)
- ☐ More than 20 years (5)

14. In which type of library do you work? Select the choice that most closely describes your library.
- ☐ Hospital library (1)
- ☐ General academic library (2)
- ☐ Academic health sciences library (3)
- ☐ Public library (4)
- ☐ Other (please describe) (5) ________________________________

15. Are you a member of professional library organizations?
- ☐ Yes (1)
- ☐ No (2)

16. Which professional library organizations are you a member of? Select all that apply.
- ☐ Medical Library Association (MLA) (1)
- ☐ A regional chapter of the Medical Library Association (2)
- ☐ Special Libraries Association (SLA) (3)
- ☐ Association for Information Science and Technology (ASIST) (4)
- ☐ American Medical Informatics Association (AMIA) (5)
- ☐ American Library Association (ALA) (6)
- ☐ Association of College & Research Libraries (ACRL) (7)
- ☐ State or regional library associations (8)
- ☐ Other (9)
